# Enhanced assembly of bacteriophage T7 produced in cell-free reactions under simulated microgravity

**DOI:** 10.1038/s41526-024-00378-4

**Published:** 2024-03-15

**Authors:** François-Xavier Lehr, Bruno Pavletić, Timo Glatter, Thomas Heimerl, Ralf Moeller, Henrike Niederholtmeyer

**Affiliations:** 1 Max Planck Institute for Terrestrial Microbiology, Marburg, Germany; 2 Center for Synthetic Microbiology (SYNMIKRO), Philipps-Universität Marburg, Marburg, Germany; 3 German Aerospace Center, Institute of Aerospace Medicine, Aerospace Microbiology, Cologne, Germany; 4 Technical University of Braunschweig, Faculty of Life Sciences, Braunschweig, Germany; 5 Technical University of Munich, Campus Straubing for Biotechnology and Sustainability, Straubing, Germany

**Keywords:** Molecular biology, Biochemistry

## Abstract

On-demand biomanufacturing has the potential to improve healthcare and self-sufficiency during space missions. Cell-free transcription and translation reactions combined with DNA blueprints can produce promising therapeutics like bacteriophages and virus-like particles. However, how space conditions affect the synthesis and self-assembly of such complex multi-protein structures is unknown. Here, we characterize the cell-free production of infectious bacteriophage T7 virions under simulated microgravity. Rotation in a 2D-clinostat increased the number of infectious particles compared to static controls. Quantitative analyses by mass spectrometry, immuno-dot-blot and real-time PCR showed no significant differences in protein and DNA contents, suggesting enhanced self-assembly of T7 phages in simulated microgravity. While the effects of genuine space conditions on the cell-free synthesis and assembly of bacteriophages remain to be investigated, our findings support the vision of a cell-free synthesis-enabled “astropharmacy”.

## Introduction

Long-term space missions will put astronaut health at immense risk^1^. Healthcare options will be limited by payload constraints and drug stability. Additionally, close quarters, radiation, and microgravity strain human health, for example by compromising the immune system^2^. On-demand production of therapeutics presents a solution to adapt medical care to the special challenges of space travel^3^. This vision of an “astropharmacy” could be realized by onboarding light weight cell-free transcription-translation (TXTL) reagents capable of rapidly synthesizing RNA and proteins from DNA blueprints, just as required. Relying only on isolated biochemical components that can be freeze-dried for storage, TXTL systems combine simplicity and flexibility, while reducing the need for downstream processing^4–6^ and biocontainment in planetary protection efforts^7^. TXTL technology enables the synthesis of large, self-assembling macromolecular complexes such as bacteriophages^8,9^ and virus-like particles^10^. Bacteriophages, as viruses that specifically target bacteria, are promising tools in the fight against multi-resistant bacteria^8,11,12^. Additionally, engineered virus-like particles could be used for gene therapy, drug delivery, and other personalized therapeutic applications^13^.

Many biological processes as well as biomolecular self-assembly depend on gravity. For example, in microgravity, proteins form larger crystals with fewer defects^14^, and amyloid fibrils nucleate and grow differently in simulated microgravity^15,16^. Comparing virus assembly in orbiter flight studies against ground controls, polyomavirus assembled into larger and more homogeneous capsomeres but did not form capsid-like structures^17^. It is hypothesized that the altered and often improved self-assembly in the absence of gravity is due to abolished sedimentation and changes in molecular transport from a convection-dominated into a diffusion-dominated regime^18^. In microorganisms, microgravity leads to alterations in cellular phenotypes and gene expression that have been observed in spaceflight samples as well as in simulated microgravity in ground-based experiments^19–21^. Microbial adaptations to the microgravity environment include changes to metabolism, host-interactions, cellular morphology as well as increased virulence and antibiotic resistance^22–24^. While the effects of microgravity on molecular self-assembly and the physiology of living organisms have been studied extensively, our understanding of the influence of microgravity on biochemical reactions is limited. Enzyme kinetics of isocitrate lyase were not affected by gravity^25^, while the catalytic efficiency of lipoxygenase-1 was four-fold higher in parabolic flight experiments^26^. CRISPR-Cas12a-mediated genetic diagnostic tests performed almost equally well onboard the International Space Station as in ground controls^27^. Conversely, DNA polymerase in vitro became more error-prone in microgravity generated by parabolic flight^28^. Despite the potential of cell-free synthesis for on-demand biomanufacturing, the influence of microgravity on TXTL systems is just beginning to be investigated. Recently, RNA-aptamers and fluorescent proteins have been successfully expressed in TXTL systems aboard the International Space Station^29^.

**Fig. 1:**
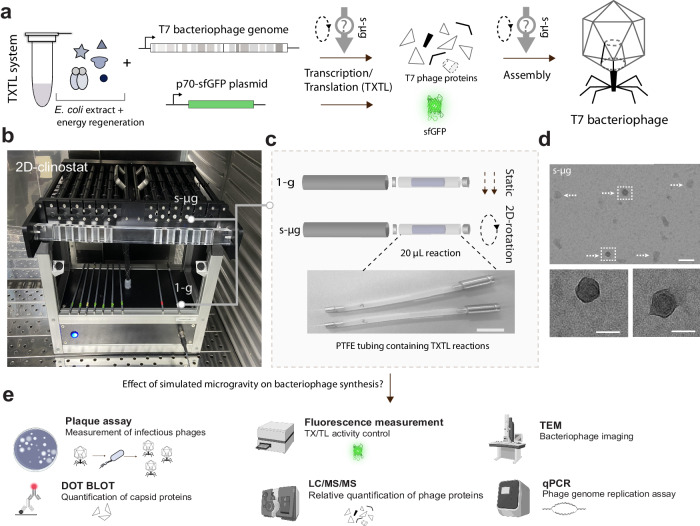
Experimental pipeline for testing the effect of simulated microgravity on the synthesis and assembly of bacteriophages in transcription-translation (TXTL) systems. **a** Phage DNA is mixed with the TXTL components to start the reaction to produce bacteriophage T7. **b** A 2D-clinostat is used to simulate microgravity (s-µg). **c** TXTL reactions are performed in Polytetrafluoroethylene (PTFE) tubes fitted to the clinostat. Control reactions are performed in identical reaction tubes held static (1-g). Scale bar: 1 cm. **d** Transmission electron microscopy (TEM) of T7 phages from the s-µg condition. Arrows indicate full size bacteriophage particles; boxes indicate phages enlarged below. Scale bars: 150 nm (top) and 50 nm (bottom). **e** Quantitative methods are used to assay protein and DNA content in TXTL samples (Dot-blot, mass spectrometry, fluorescence measurements, real-time quantitative PCR) and the assembly of infectious bacteriophages (plaque assays, TEM).

Here we focus on the cell-free production of the model bacteriophage T7 under simulated microgravity (s-µg) to investigate the effects of s-µg on a complex biochemical reaction involving synthesis and self-assembly processes. 2D-clinorotation approximates microgravity by rotating a sample to prevent sedimentation and has been used to simulate microgravity in experiments with different cell types and organisms^30–33^. Bacteriophage T7 infects *Escherichia coli* bacteria and has a 40 kb genome, encoding 56 proteins. Each infectious phage particle is assembled from a total number of roughly 500 protein subunits and a DNA molecule that is tightly packed into the capsid^34^. In a synthesis reaction containing purified T7 DNA and TXTL reagents, the number of infectious phage particles produced depends on three major processes: transcription, translation, and self-assembly (Fig. 1a). By quantifying the effects of simulated microgravity on cell-free synthesis and self-assembly of a complex multi-protein structure, our work takes an initial step in assessing the possibility of TXTL-assisted on-demand manufacturing of biologics in space.

## Results and discussion

### Higher counts of plaque forming units from synthesis reactions in s-µg

**Fig. 2:**
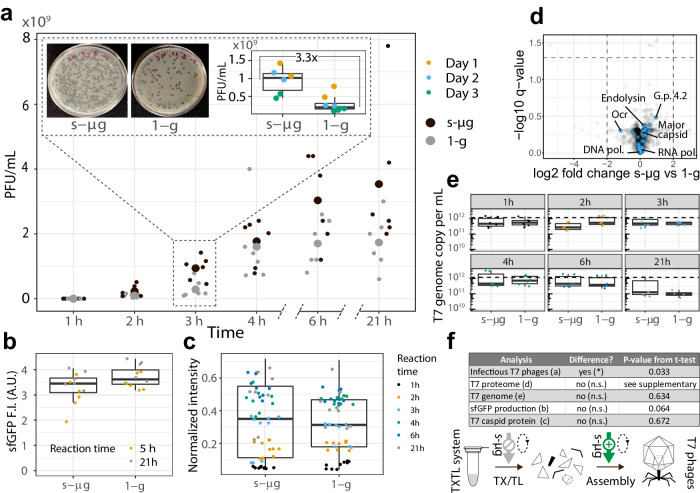
Quantitative analyses of T7 bacteriophage production in s-µg and 1-g conditions. **a** Increased T7 phage production in s-µg. Plaque forming units were determined at six different timepoints of the cell-free reaction. A zoom on the 3 h timepoint shows images of plaque assay plates along with the measurements color-coded by day of experiment. **b** Production of sfGFP in control reactions set up in parallel to phage production. **c** Relative quantification of the T7 major capsid protein by immuno-dot-blot. **d** Proteomic analysis showed no significant differences in phage protein contents between the two conditions at 21 h. Volcano plot comparing s-µg against the 1-g condition. Gray dots represent the *E. coli* proteome and the blue dots correspond to T7 phage proteins with names of selected T7 proteins. **e** Quantification of the T7 genome copy number by qRT-PCR showed no effect of s-µg on T7 genome copy number. The dashed line indicates the initial number of T7 DNA molecules in the samples (1.204 × 10^12^ per mL, 2 nM). **f** Summary of the performed analyses, their results and statistical significance. No significant (n. s.) differences were observed in concentrations, suggesting enhanced assembly. The midlines of the boxplots represent the median, and the boxes’ upper and lower limits represent the 1^st^ and 3^rd^ quartiles.

To accommodate TXTL reactions in the 2D-clinostat, we customized vessels to support the small volumes (10–100 µL) typically used in cell-free protein synthesis. Briefly, polytetrafluoroethylene (PTFE) tubing was enclosed into polyvinyl chloride (PVC) tubes fitting the clinostat rotational tray (Fig. 1b, c). We first verified synthesis and assembly of T7 bacteriophages were possible and reproducible in the PTFE vessels. Compared to standard 1.5 mL microreaction tubes, we observed a slight decrease of plaque forming units (PFU) per mL (Supplementary Fig. 1). The synthesis yields of TXTL reactions are influenced by oxygen availability, as it plays a crucial role in facilitating energy regeneration through oxidative phosphorylation^35^. Hence, we hypothesize that the observed decline in PFU can be attributed to the increased volume-to-air interface ratio in the adapted vessels compared to the standard microreaction tubes with equivalent reaction volumes^36^. Nevertheless, the low variability observed in the adapted vessels enabled us to compare phage synthesis kinetics in clinorotation and static control conditions. A TXTL mastermix containing 2 nM of T7 DNA was split into samples for timepoints for s-µg and stationary control (1-g) conditions. For each timepoint, plaque assays were performed in duplicates. The experiment was repeated on three consecutive days (Supplementary Fig. 2). The kinetics of phage synthesis were similar in s-µg and 1-g conditions (Fig. 2a), and comparable to reactions in standard gravity and reaction tubes^9^. After two hours, plaques were detected in both conditions, and phage yield increased until 6 h of incubation, after which phage yields plateaued. We observed a consistently higher number of phages in s-µg for all productive timepoints with the highest difference in PFU after three hours of incubation. At 3 h, on average, 3.3-times as many PFU were counted in s-µg (Fig. 2a). Considering the low strength of the gravitational force on biomolecular systems with low sedimentation rates, the observed difference is notable and comparable in magnitude to the effects of microgravity on other biochemical reactions^26,28^. Statistical analysis performed on all timepoints combined (unpaired t-test, *p*-value = 0.033) also confirmed a significantly higher number of synthesized phages in s-µg compared to 1-g (Supplementary Fig. 3).

We used transmission electron microscopy to confirm bacteriophage production. Typical icosahedral capsids of bacteriophage T7 were successfully visualized (Fig. 1d). Notably, TEM samples with bacteriophages produced in s-µg contained a higher number of fully assembled phage capsids compared to 1-g samples. Conversely, fewer small, incomplete assemblies were visible in s-µg compared to 1-g (Supplementary Figs. 4 and 5).

### S-µg environment likely enhances bacteriophage self-assembly

Since we measured an increased number of infectious phages in the s-µg environment, we next asked if the s-µg conditions enhanced synthesis reactions or phage assembly. A parallel TXTL reaction producing superfolder green fluorescent protein (sfGFP) did not show a significant difference in fluorescence intensity after 5 or 21 h of incubation between s-µg and 1-g (Fig. 2b, unpaired t-test, *p* = 0.064). This suggests that the enhanced number of phages obtained in s-µg may not be the result of enhanced transcription or translation, but may rather be due to improved phage assembly. To support this hypothesis, we compared the composition of TXTL reactions from both conditions with several additional quantitative methods.

To gain insights in the phage protein production along the kinetics, we performed immuno-dot-blot assays targeting the major capsid protein, the most abundant structural T7 phage protein (Fig. 2c). Over the time course of the experiments, we did not find an overall significant difference (unpaired t-test, *p*-value = 0.672) in capsid protein signal between s-µg and 1-g conditions. To extend our analysis to the entire T7 proteome, the protein content of the 21 h endpoint samples was analyzed by mass spectrometry. Over 1000 *E. coli* proteins were detected in each preparation and, as expected, showed similar composition in all samples because our TXTL reagents consist of an *E. coli* lysate (Supplementary Fig. 6a). In total, 80% of the bacteriophage T7 proteome was detected in s-µg and 1-g, including hypothetical (e.g. gene product 4.2), structural (e.g. major capsid protein) and non-structural proteins (e.g. DNA polymerase) (Supplementary Fig. 6b). Using t-tests and q-values adjusted for false discovery rate, we did not observe significant differences between s-µg and 1-g for the 45 detected phage proteins (Fig. 2d, Supplementary Table 1). Hierarchical clustering of the detected T7 proteins did not lead to any visible patterns (Supplementary Fig. 7). The proteomics results therefore support our previous findings that s-µg did not alter protein synthesis (Fig. 2f).

The bacteriophage T7 genome has been previously shown to replicate in TXTL reactions^37^. We hypothesized that the higher number of infectious phages in s-µg could stem from differences in genome replication or DNA degradation. To measure if s-µg samples contained higher amounts of DNA, we used quantitative real-time PCR (qRT-PCR) to track the bacteriophage T7 genome copy number over time^38^ (Fig. 2e). Similar to our protein content comparisons, no significant difference was observed in the DNA copy numbers between s-µg and 1-g conditions (one-way ANOVA, *p*-value = 0.223), indicating that the higher phage yield in s-µg was not a result of differences in DNA content. We observed no significant changes in the DNA concentration over time (one-way ANOVA, *p*-value = 0.0966), indicating no replication in s-µg or 1-g, possibly due to our high starting DNA concentration. Alternatively, replication and degradation of the linear DNA template may have been balanced.

### Robustness to changes in experimental parameters

While the main focus of our study was on the impact of simulated microgravity on TXTL reactions, future research could explore the influence of aeration on TXTL reactions in s-µg conditions, which could be performed in high-aspect ratio vessels (HARV)^19,39,40^ after their adaption to small reaction volumes. Conversely, the adaptions we made to the 2D-clinostat vessels are valuable to study the impact of additional factors that could either stimulate or inhibit the observed enhanced assembly. These factors include variations in buffer components and temperature, as well as the lysate itself, which is known to display a high batch-to-batch variability^41^. Parameters like these have been shown to impact cell-free synthesis and phage production yields in a static environment^9,42^. We tested the effects of s-µg at such varying conditions to determine the robustness of our results. Guided by our previous data, we performed plaque assays after 3 h of TXTL synthesis, i.e. when we expected the highest fold-change in PFU. While the variation of experimental parameters affected the absolute PFU count, enhanced T7 bacteriophage production in s-µg remained robust to most of these variations. For example, we successfully replicated the enhanced production of T7 bacteriophages in s-µg conditions using a new batch of TXTL reagents under standard reaction conditions (Supplementary Fig. 8a). TXTL reactions with *E. coli* lysate are typically performed at 29 °C for optimal protein synthesis. However, even at 37 °C, the optimal temperature for *E. coli* host growth and T7 bacteriophage infection, the PFU titer in s-µg conditions was 2.03-times higher than in 1-g (*p*-value = 0.0097) (Supplementary Fig. 8b). In addition to temperature, the concentration of crowding agent (PEG-8000 in our TXTL reactions) is crucial for cell-free phage production, as molecular crowding favors self-assembly^9^. In the batch of TXTL reagents used for the experiments in Fig. 2, the optimal PEG-8000 concentration was 3.5% (v/v), serving as the standard condition. In the new batch, with a lower PEG-8000 concentration of 2.5% (v/v), 4.1-times as many PFU were counted on average (*p*-value = 0.003), while at 4.5% (v/v) the higher average PFU in s-µg was not statistically significant (Supplementary Fig. 8c), which may be due to a lower mixing efficiency during clinorotation at increased viscosity.

Moreover, it will be interesting to study bacteriophages beyond T7 with higher (e.g. T4) or lower (e.g. MS2) complexities. The growing number of cell-free biotechnological tools enabling quick manipulation of phage gene expression^43^ and transient phage engineering^8^ could be used to further explore phage assembly in microgravity by altering expression of structural genes in the TXTL reaction.

## Summary

In summary, our results show enhanced bacteriophage yields in simulated microgravity by 2D-clinorotation. Our quantitative analyses of protein and DNA contents revealed no differences between s-µg and 1-g samples. Since proteins and DNA are the building materials for virion assembly, we conclude that the increase in infectious bacteriophage particles is most likely due to enhanced self-assembly of bacteriophage T7 in simulated microgravity, as previously shown for protein crystal assembly in microgravity^14^. In 2D-clinostat experiments, the gravity vector changes continuously and effectively cancels out due to the rotation around a horizontal axis. We hypothesize that the reduction of sedimentation in simulated microgravity explains the improved self-assembly. According to this hypothesis, orbital shaking experiments could also be expected to improve phage yields because shaking in a horizontal plane reduces sedimentation as well, even though neither clinorotation nor orbital shaking lead to movement of the liquid body or the water-air interfaces in the sample vessels we used (Supplementary Fig. 9a, Supplementary Movie 1). Vessel architecture and small reaction volumes allowed us to minimize potential contributions from moving air-water interfaces or hydrodynamic mixing that have been shown to influence amyloid fibril assembly in larger reaction volumes^16^. Similarly to 2D-clinorotation, orbital shaking experiments also yielded increased PFU counts and support our hypothesis that preventing sedimentation improves bacteriophage production (Supplementary Fig. 9b, c). To enhance Earth-based cell-free production of other potential therapeutics that rely on self-assembly, it may be beneficial to prevent sedimentation by clinorotation or shaking.

Our results indicate that microgravity conditions might improve cell-free biomanufacturing yields, which is encouraging for the vision of an “astropharmacy” enabled by cell-free synthesis. Looking ahead, freeze-drying TXTL systems^4,44,45^ presents a promising avenue to facilitate storage and transportation^46^, especially under the challenging conditions encountered during space missions. An important next step could involve testing the impact of additional spaceflight conditions such as increased ionizing radiation on cell-free synthesis. Together with microgravity, radiation represents a main feature of the spaceflight environment, impacting astronaut health and stability of biomolecules^47^. Efforts to understand cell-free synthesis mechanics in spaceflight conditions will advance the biomanufacturing and therapeutic options available for future space exploration.

## Methods

### TXTL extract preparation

TXTL systems preparation was adapted from Silverman et al.^41^, including adaptions described by Falgenhauer et al.^48^. *E. coli* BL21 Rosetta 2 were streaked overnight on an agar plate containing chloramphenicol. One colony was picked and inoculated overnight in 50 mL 2xYT supplemented with chloramphenicol for growth at 37 °C. After a minimum of 15 hours, 20 mL of the stationary culture was used to inoculate 400 mL of 2xYT + P media (16 g/L tryptone, 10 g/L yeast extract, 5 g/L sodium chloride, 7 g/L potassium phosphate dibasic, 3 g/L potassium phosphate monobasic) in a 1 L baffled flask. Cells were grown at 40 °C and 200 RPM to 3.0 ± 0.2 OD_600_. Centrifuge bottles were filled up to 300 mL and centrifuged for 10 minutes at 4000 × *g* at 4 °C and supernatants were discarded. The pellets were washed three times with 25 mL buffer S30A (50 mM Tris-base, 14 mM Mg-glutamate, 60 mM K-glutamate, 2 mM DTT, brought to pH 7.7 with acetic acid). The washing steps were followed by a centrifugation step at 4000 × *g* at 4 °C for 10 min. A fourth centrifugation step at 3000 × *g* at 4 °C for 10 min enabled the removal of the remaining traces of buffer. The pellets were then resuspended in 1 mL of Buffer S30A per gram of pellet and supplemented with 0.5 mg per mL of lysozyme (from chicken egg, >40,000 units per mg, Sigma). The resuspended pellets were incubated for 10 min on ice. 1 mL of the suspension was aliquoted into 1.5 mL Eppendorf tubes. The pellet suspensions were then lysed with a sonicator (QSonica Q125 with a 3.175 mm diameter probe, 50% amplitude, 20 kHz, and 10 s ON/OFF pulses). Each sample was sonicated until reaching 250 J input. Using a 100 mM stock solution, 1 mM of DTT was added to each crude lysate immediately after sonication. The cell lysate was centrifuged for 10 min at 4 °C and 12,000 × *g*. The supernatant was removed and placed into an incubator set up at 37 °C and 200 RPM for 80 min. After the run-off reaction, the supernatant was centrifuged for 10 min at 4 °C and 12,000 × *g*. Finally, the extract was dialyzed for 3 h against buffer S30B (50 mM Tris-base, 14 mM Mg-glutamate, 60 mM K-glutamate, 2 mM DTT, pH 8.2) in a 10k MWCO cassette (Thermofisher). Finally, the dialyzed extract was centrifuged for 10 min at 4 °C and 12,000 × *g*. The supernatant was aliquoted, snap-frozen into liquid nitrogen, and stored at −80 °C.

### TXTL mastermix reaction

The final TXTL reaction mixture is composed of the following reagents: 33% v/v of *E. coli* extract, 10 mM ammonium glutamate; 1.2 mM ATP; 0.850 mM each of GTP, UTP, and CTP; 0.034 mg per mL folinic acid; 0.175 mg per mL yeast tRNA; 2 mM amino acids; 30 mM 3-PGA; 0.33 mM NAD; 0.27 mM CoA; 1 mM putrescine; 1.5 mM spermidine; 4 mM oxalic acid; 57 mM HEPES, 3.5% PEG 8000, 5 µM of Chi6 linear DNA. Mg-glutamate and K-glutamate were optimized for phage production and set respectively to 7 mM and 170 mM. T7 Phage genomic DNA (Bioron) was set to 2 nM (1.204 × 10^12^ DNA molecules per mL).

### Clinostat operation and TXTL loading

The 2D-clinostat used in this study was developed and kindly provided by the DLR Institute of Aerospace Medicine, Department of Gravitational Biology and has been used in previous studies on the effects of microgravity on biological systems. The clinorotation speed was 60 RPM. The TXTL mastermix was prepared with all components except T7 Phage genomic DNA. The mastermix was split into 20-µL aliquots, and each was introduced inside its own PTFE tube (1/16“ID x 1/8“OD). The PTFE tubes were sealed with metal caps (Fig. 1c). The T7 Phage genomic DNA was added just before loading the PTFE tubes, to minimize the reaction time before the experiment. Instead of T7 DNA, negative controls contained nuclease-free water, and translation control tubes contained a plasmid expressing sfGFP under the control of a strong constitutive promoter. For the samples analyzed with proteomics, the loaded volume was 50 µL. The PTFE tubes were inserted into hollow PVC tubes fitting the clinostat rotational tray. The clinostat containing all the tubes was held in an incubator at 29 °C. The samples of TXTL were taken after 1 h, 2 h, 3 h, 4 h, 6 h, and 21 h of incubation. For each timepoint, plaque assays were performed in duplicate with serial dilutions by plating samples immediately. Static and orbital shaking control experiments were performed in the same PTFE tubing sealed with metal caps, and at the same volume as 2D-clinorotation samples.

### Phage infectivity assay

At each timepoint, serial decimal dilutions of the TXTL reactions were prepared in Luria-Bertani (LB) medium. Then, 5 µL of each serial dilution was mixed with 130 µL of *E. coli* DSM 613 (OD_600_ between 0.8 and 1) and incubated for 3 min at 37 °C. All 135 µL were transferred into 1.75 mL of soft LB medium, mixed well, and poured onto a 60 mm petri dish. Each petri dish was left for 30 min at room temperature to solidify and then transferred to 37 °C. The plaques were counted after 3 h of incubation. After preparing the serial dilutions for the infectivity assays, samples were frozen at −80 °C with 25% (v/v) glycerol until used for other analysis of protein content, replication, and assembly.

### sfGFP fluorescence

sfGFP fluorescence was measured after 5 and 21 h of incubation. The TXTL reactions contained a plasmid expressing sfGFP under the control of a strong constitutive *E. coli* promoter. The fluorescence intensity (excitation: 485 nm; emission: 515 nm) was measured with a plate-reader (TECAN Infinite M200 Pro) and compared between s-µg and 1-g samples (10 µL of the reaction sample were used).

### Transmission electron microscopy

30 µL of the diluted TXTL samples from the 21 h timepoint was further diluted to 200 µL with TBS (Tris 20 mM, NaCl 150 mM, pH 7.6). The samples were extracted by adding 10 µL of chloroform and centrifuged for 10 min at 13,000 × *g*. The upper aqueous phase was transferred to a new tube. The bacteriophages were precipitated by adding 50 µL of PEG/NaCl 5x buffer (PEG-8000 20%, NaCl 2.5 M). The tubes were incubated at 4 °C overnight. The bacteriophages were pelleted by centrifugation for 10 minutes at 13,000 × *g*. The bulks of supernatants were removed. The samples were centrifuged again for 10 minutes at 13,000 × *g* and the supernatant was completely removed. The pellets were resuspended in 20 µL TBS. Carbon-coated copper grids (400 mesh) were hydrophilized by glow discharging (PELCO easiGlow, Ted Pella, USA). 5 µL of the primary antibody solution (T7 tag, PA1-32386, Thermofisher) was applied onto the hydrophilized grids. After 2 min of incubation, the solutions were briefly blotted and 5 µL of the samples were applied onto the grids. Alternatively, for Supplementary Figs. 4 and 5, “antibody coated” grids were incubated in diluted sample solution (1:40) for 1 h under shaking. After two short washing steps in droplets of double distilled water, samples were stained with 2% uranyl acetate. Samples were analyzed with a JEOL JEM-2100 transmission electron microscope using an acceleration voltage of 120 kV. Images were acquired with a F214 FastScan CCD camera (TVIPS, Gauting).

### Proteomics

Proteins contained in TXTL samples (50 µL) were reduced by adding 5 mM Tris(2-caboxyethyl)phosphine at 90 °C for 15 min, followed by alkylation (10 mM iodoacetamide, 30 min at 25 °C). The amount of extracted proteins was measured using BCA protein assay (Thermo Fisher Scientific). 50 µg total protein was then digested with 1 µg trypsin (Promega) overnight at 30 °C in the presence of 0.5% SLS. Following digestion, SLS was precipitated with trifluoroacetic acid (TFA, 1.5% final concentration) and peptides were purified using Chromabond C18 microspin columns (Macherey-Nagel). Acidified peptides were loaded on spin columns equilibrated with 400 µL acetonitrile and then 400 µL 0.15% TFA. After peptide loading, a washing step with 0.15% TFA was performed, followed by elution using 400 µL 50% acetonitrile. Eluted peptides were then dried by vacuum concentrator and reconstituted in 0.15% TFA.

Peptide mixtures were analyzed using liquid chromatography-mass spectrometry carried out on an Exploris 480 instrument connected to an Ultimate 3000 RSLC nano with a Prowflow upgrade and a nanospray flex ion source (all Thermo Scientific). Peptide separation was performed on a reverse phase HPLC column (75 μm × 42 cm) packed in-house with C18 resin (2.4 μm, Dr. Maisch). The following separating gradient was used: 94% solvent A (0.15% formic acid) and 6% solvent B (99.85% acetonitrile, 0.15% formic acid) to 25% solvent B over 40 min and to 35% B for additional 20 min at a flow rate of 300 mL per min. DIA-MS acquisition method was adapted from Bekker-Jensen et al. ^49^. In short, Spray voltage were set to 2.0 kV, funnel RF level at 55, and heated capillary temperature at 275 °C. For DIA experiments full MS resolutions were set to 120.000 at m/z 200 and full MS AGC target was 300% with an IT of 50 ms. Mass range was set to 350–1400. AGC target value for fragment spectra was set at 3000%. 45 windows of 15 Da were used with an overlap of 1 Da. Resolution was set to 15,000 and IT to 22 ms. Stepped HCD collision energy of 25, 27.5, 30% was used. MS1 data was acquired in profile, MS2 DIA data in centroid mode. Analysis of DIA data was performed using DIA-NN version 1.8^50^ using Uniprot protein databases from *E. coli* and bacteriophage T7. Full tryptic digest was allowed with two missed cleavage sites, and oxidized methionines and carbamidomethylated cysteines. Match between runs and remove likely interferences were enabled. The neural network classifier was set to the single-pass mode, and protein inference was based on genes. Quantification strategy was set to any LC (high accuracy). Cross-run normalization was set to RT-dependent. Library generation was set to smart profiling. DIA-NN exports were further statistically evaluated using a modified SafeQuant script made compatible to process DIA-NN data.

### Dot-Blot

2 µL of the diluted TXTL samples were blotted on a nitrocellulose membrane and let dry for 15 minutes. The membrane was incubated for 1 h with blocking buffer (TBST 1×, 5% w/v non-fat dry milk) on a shaker at RT. The membrane was rinsed 2 times with water and incubated for 1 hour on a shaker at room temperature (RT) with the primary antibody (T7 tag, PA1-32386, Thermofisher) diluted in the blocking buffer (1:10,000). The membrane was washed 3 times with the wash buffer (TBST 1×) and incubated for 1 h on a shaker at RT with the secondary antibody (IRDye® 800CW Goat anti-Rabbit IgG, LI-COR) diluted (1:20,000) in the blocking buffer. Finally, the membrane was washed 3 times with the wash buffer and scanned for fluorescence with an LI-COR Odyssey DLx. The calibration curve showed that the concentration of synthesized phages from the experiment was in the linear range of detection (Supplementary Fig. 10). For each experiment day (*n* = 3), the time points for the s-µg and the 1-g conditions were blotted on the same membrane along with an internal standard (bacteriophage T7 synthesized in TXTL at 1 nM DNA concentration). Dot blots of the three experiment days were performed on three consecutive days and analyzed in parallel by normalization to the internal standard.

### qRT-PCR

To determine the bacteriophage T7 DNA copy number, qRT-PCR was performed. Each TXTL sample was diluted 100x in nuclease-free water, and 5 µL of the diluted TXTL was used for qRT-PCR, as described earlier for T7 phage produced in TXTL^43^. The assays were performed in technical duplicates for each of the timepoints from all three experimental replicates. The primers and the program were adapted from Peng et al.^38^ (Supplementary Table 2, Supplementary Table 3). The kit used for the qRT-PCR reaction was NEB Luna® Universal qPCR & qRT-PCR. As a standard, 5 µL of T7 Phage DNA (Bioron) was used. The standard was diluted in nuclease-free water. Different dilutions were used: 0.2 nM (610,000,000 T7 DNA molecules), 0.2 × 10^−2^ nM (6,100,000 T7 DNA molecules), 0.2 × 10^−4^ nM (61,000 T7 DNA molecules), and 0.2 × 10^-6^ nM (610 T7 DNA molecules). The determined DNA copy number in each sample was corrected for the original TXTL reaction concentration and volume (100× concentrated, 20 µL) (Supplementary Fig. 11).

### Supplementary information

Supplementary Information

Supplementary Video 1

## Data Availability

The authors declare that all relevant data supporting the findings of this study are available within the paper and its Supplementary information files. The mass spectrometry proteomics data have been deposited to the ProteomeX change Consortium via the PRIDE partner repository with the dataset identifier PXD044176. Additional data are available from the corresponding author upon request.
