# Supplementary material for: Enhanced assembly of bacteriophage T7 produced in cell-free reactions under simulated microgravity
Source: NPJ Microgravity. 2024 Mar 15;10:30. doi: 10.1038/s41526-024-00378-4 (PMC10943216; doi:10.1038/s41526-024-00378-4)
Supplement: Supplementary file 1 — Supplementary Information [file 41526_2024_378_MOESM1_ESM.pdf]

Supplementary Information for

Enhanced assembly of bacteriophage T7 produced in cell-free reactions under simulated microgravity

François-Xavier Lehr[1,2#] & Bruno Pavletić[3,4#], Timo Glatter[1], Thomas Heimerl[2], Ralf Moeller[3*], Henrike Niederholtmeyer[1,2,5*]

[1]Max Planck Institute for Terrestrial Microbiology, Marburg, Germany

[2]Center for Synthetic Microbiology (SYNMIKRO), Philipps-Universität Marburg, Marburg, Germany

[3]German Aerospace Center, Institute of Aerospace Medicine, Cologne, Germany

[4]Technical University of Braunschweig, Faculty of Life Sciences, Universitätsplatz 2, 38106 Braunschweig, Germany

[5]Campus Straubing for Biotechnology and Sustainability, Technical University of Munich, Germany

[#]FXL and BP contributed equally to this work.

*Correspondence to:

Ralf Moeller: Ralf.Moeller@dlr.de

Henrike Niederholtmeyer: henrike.niederholtmeyer@tum.de

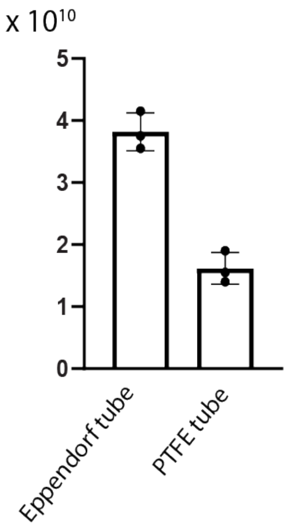

Supplementary Figure 1. Cell-free synthesis efficiency of T7 bacteriophages decreases in PTFE tubes. Plaque assay variability remains low when PTFE tubes are used as a reaction vessel.

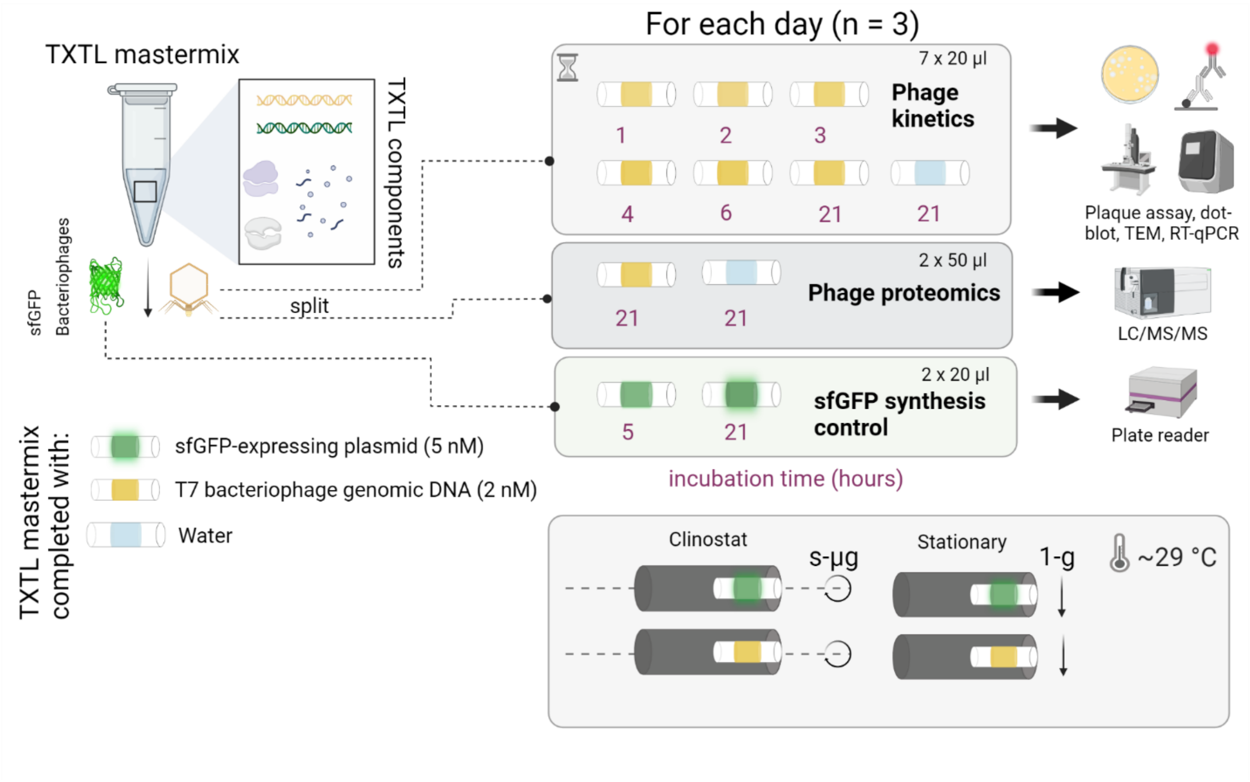

Supplementary Figure 2. Experimental set-up of the cell-free synthesis operated in simulated microgravity and in stationary control conditions. The experimental set-up shown corresponds to one day of experiment and was repeated 3 times.

a

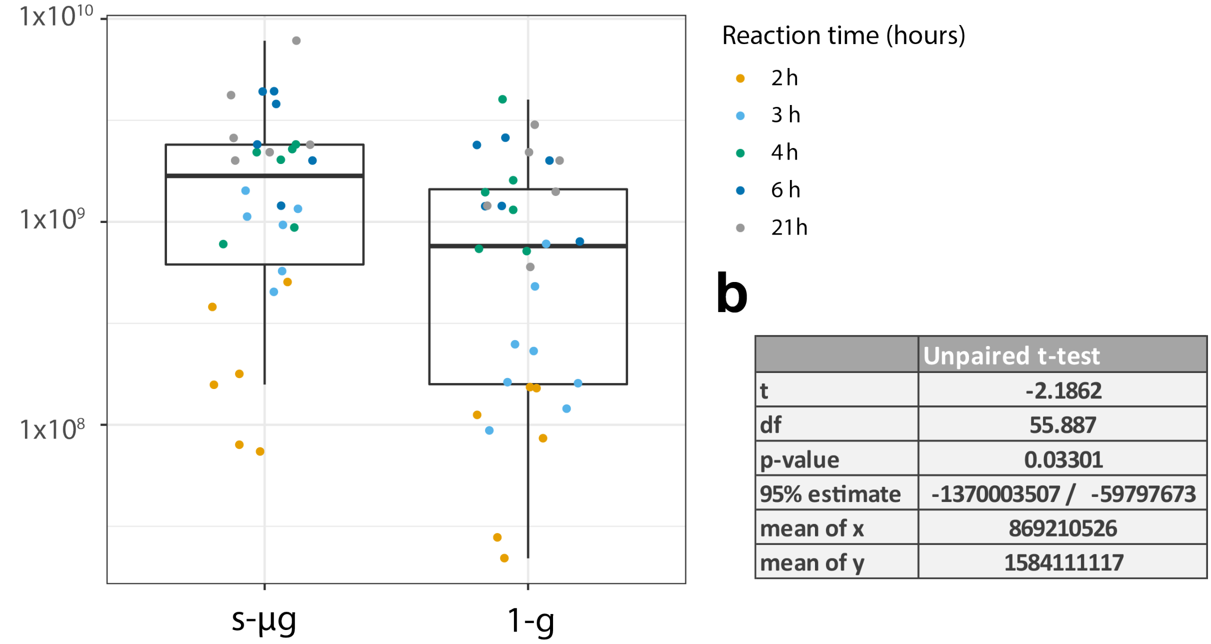

b

	Unpaired t-test
t	-2.1862
df	55.887
p-value	0.03301
95% estimate	-1370003507 / -59797673
mean of x	869210526
mean of y	1584111117

Supplementary Figure 3. a) Summary of PFU/mL of bacteriophages T7 synthesized in s-µg and 1-g. b) Statistical summary table for unpaired t-test comparing the two conditions.

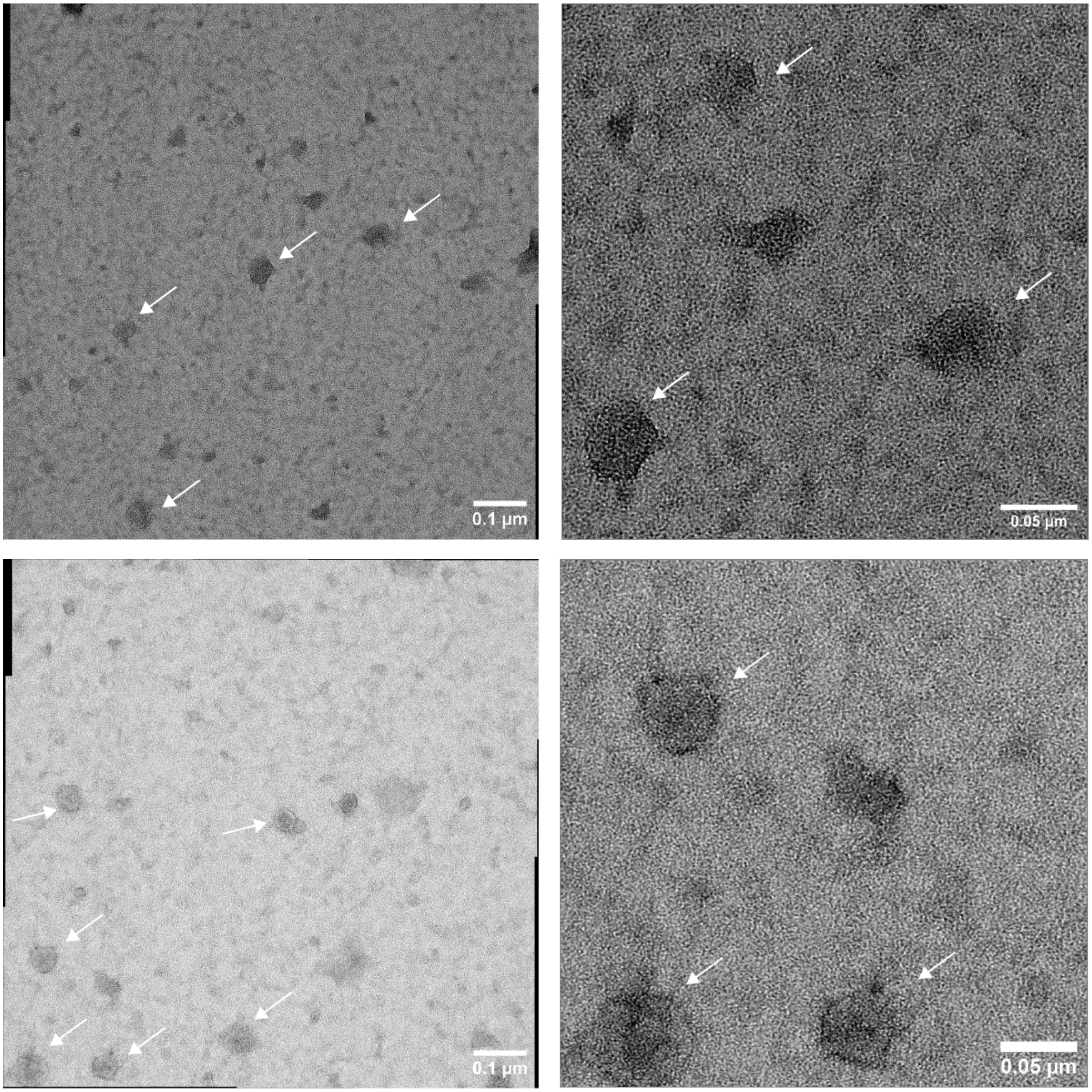

Supplementary Figure 4. T7 bacteriophages synthesized in s-µg visualized by transmission electron microscopy. White arrows indicate presumed fully assembled phage capsids.

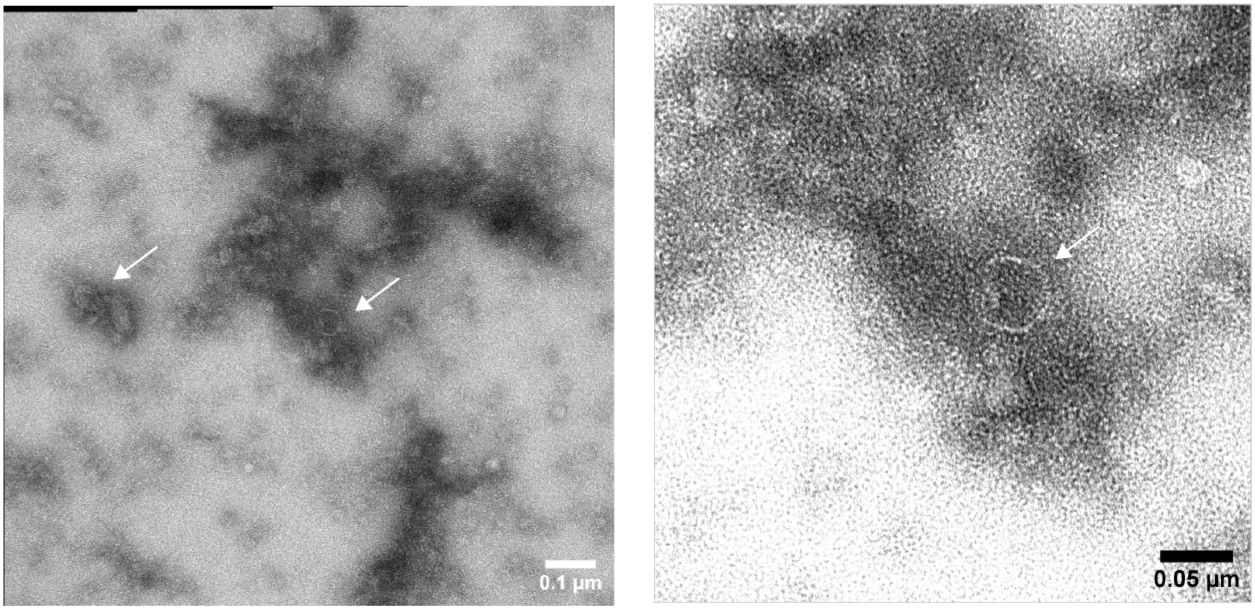

Supplementary Figure 5. T7 bacteriophages synthesized in 1-g visualized by transmission electron microscopy. White arrows indicate presumed fully assembled phage capsids.

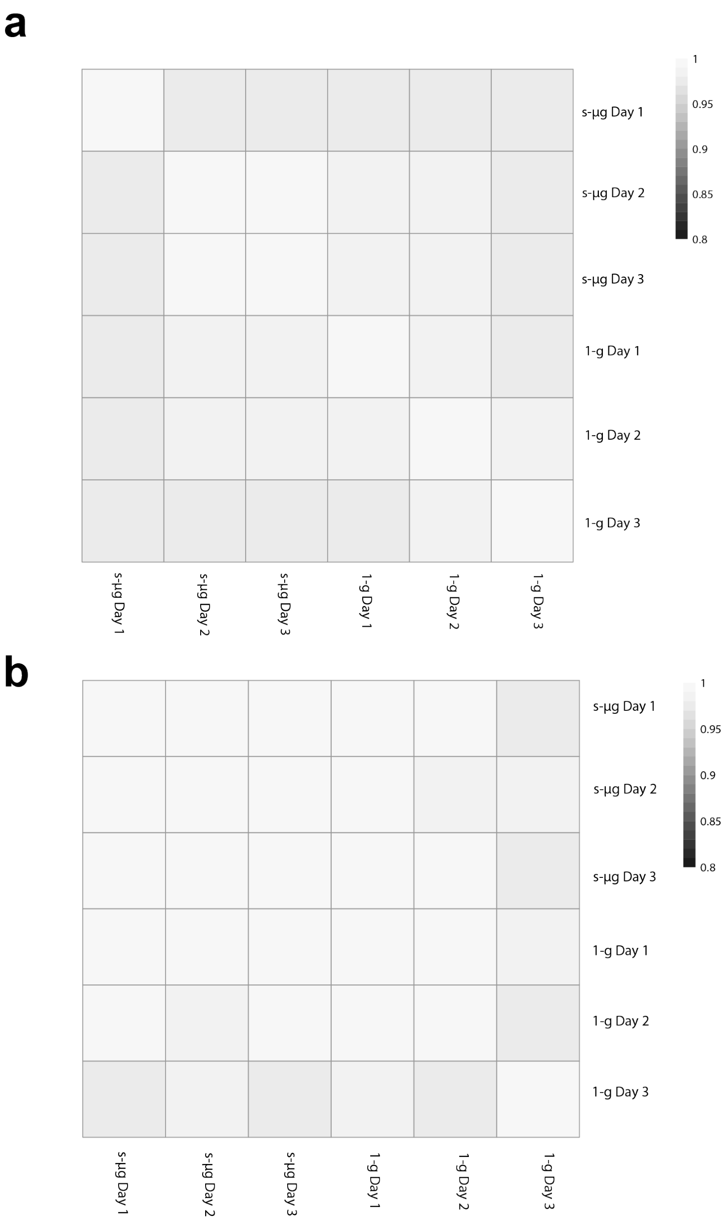

Supplementary Figure 6. Pearson correlation matrix of the detected *E. coli* proteome (a) and T7 proteins (b) between the three experimental replicates in s-µg and 1-g.

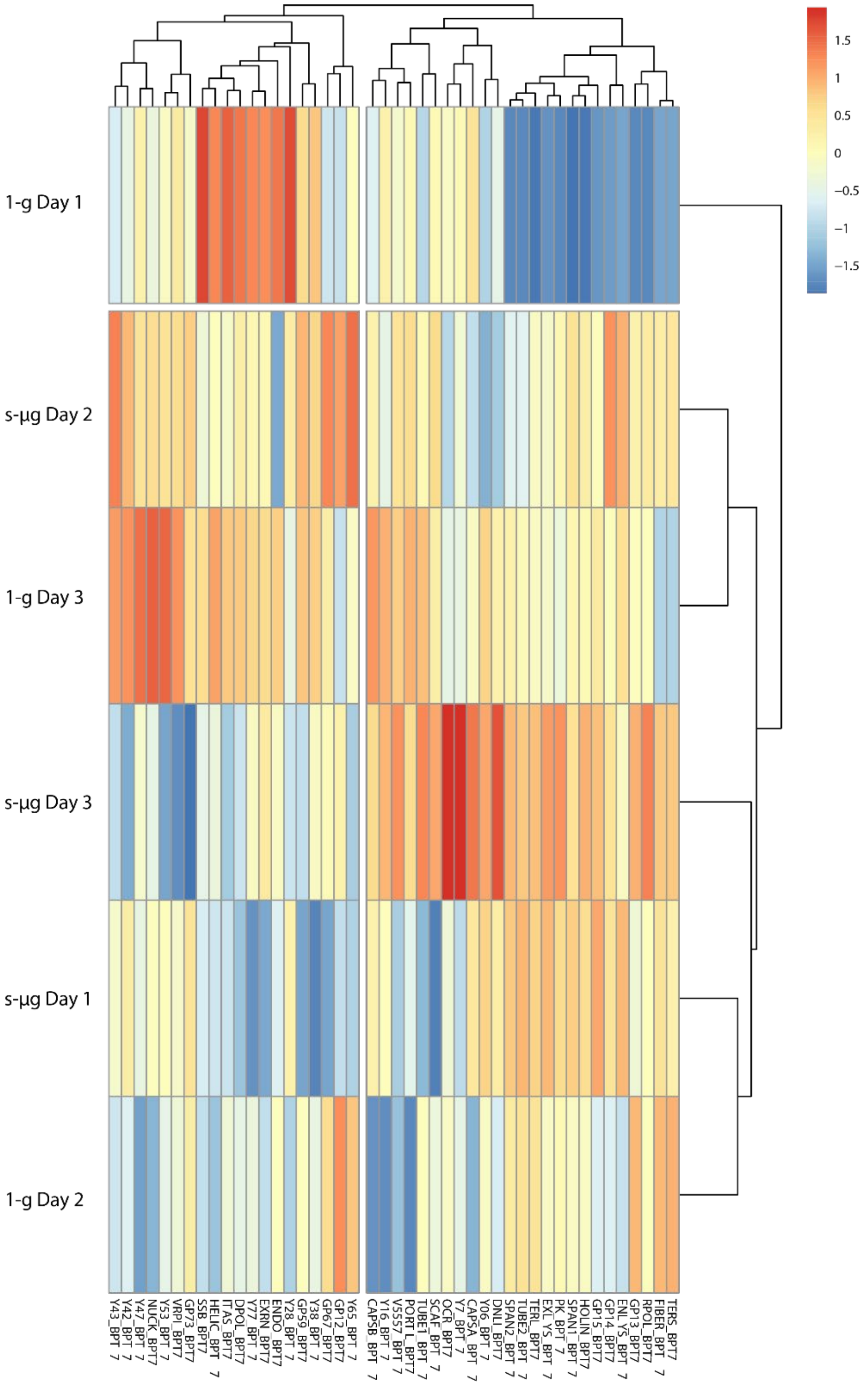

Supplementary Figure 7. Heatmap representation of the T7 bacteriophage proteins detected in 1-g and s-µg. Vertical columns represent the proteins identified by LC-MS/MS. Rows depict the biological replicates in s-µg and 1-g. The protein intensities values were centered and scaled in column direction and used for hierarchical clustering of the samples by using Euclidian distance and average method (constructed using the pheatmap package from R).

a

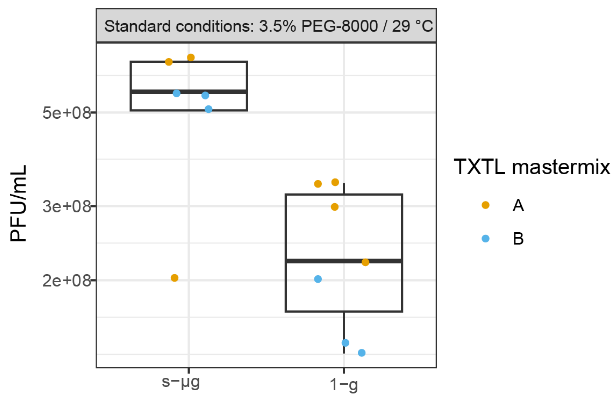

Standard	Unpaired t-test
t	-3.1982
df	4.9881
p-value	0.02412
95% estimate	-521362487 -56537513
mean of x	232250000
mean of y	521200000
fold-change	2.24

b

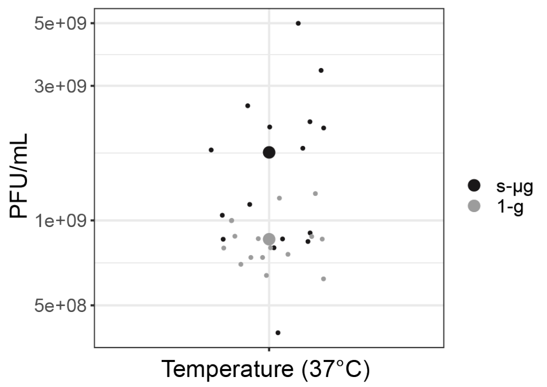

37 °C	Unpaired t-test
t	-2.9422
df	15.676
p-value	0.009727
95% estimate	-1521585128 -245914872
mean of x	857500000
mean of y	741250000
fold-change	2.03

c

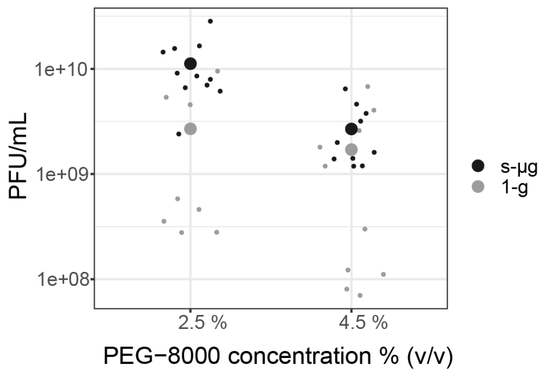

2.5 % PEG-8000	Unpaired t-test
t	-3.4193
df	15.267
p-value	0.003722
95% estimate	-13797736297 -3211263703
mean of x	2.70E+09
mean of y	1.12E+10
fold-change	4.15

4.5 % PEG-8000	Unpaired t-test
t	-1.0795
df	17.145
p-value	0.2953
95% estimate	-2870520618 926520618
mean of x	1.71E+09
mean of y	2.680e+09
fold-change	1.56

Supplementary Figure 8. Enhanced bacteriophage T7 assembly in s-µg is robust to changes in TXTL parameters. a) Bacteriophage T7 PFUs comparison after three hours of cell-free synthesis between s-µg and 1-g conditions in a different batch of TXTL with standard buffer parameters (3.5 % (v/v) PEG-8000, 29 °C incubation temperature). Data points "A" and "B" represent results from two independently prepared reaction mixes. b) Experiment described in a) was repeated with an incubation temperature of 37 °C. c) Experiment described in a) was repeated with a lower (2.5 % (v/v)) or higher (4.5 % (v/v)) concentration of PEG-8000. Thick markers represent the average, small markers show individual data points. Statistical summary tables for unpaired t-test comparing the two conditions are presented on the right.

a

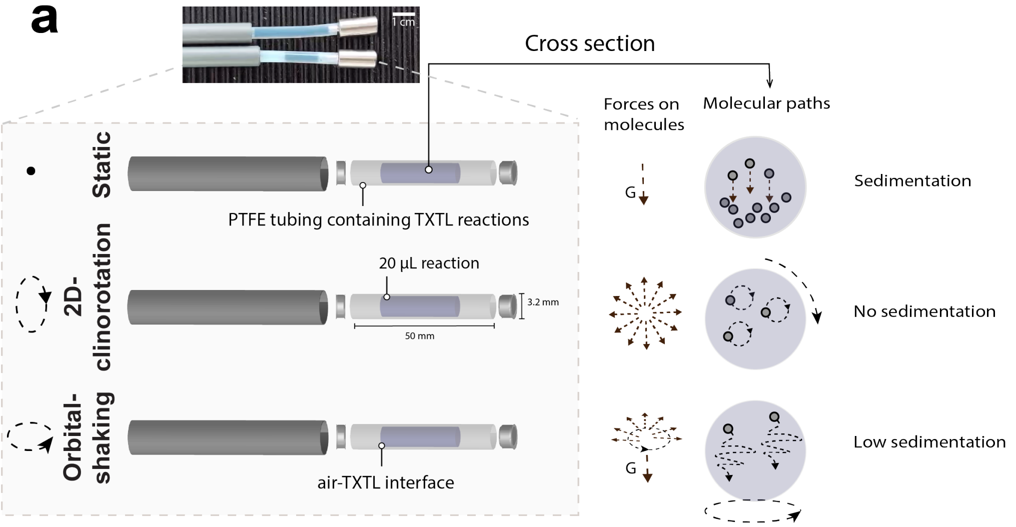

b

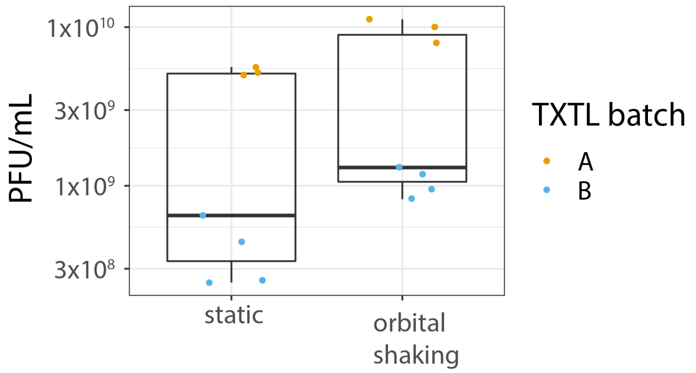

c

	Unpaired t-test
t	-2.3384
df	13.124
p-value	0.03583
95% estimate	-9578256189 / -383629525
mean of x	2484857143
mean of y	7465800000

Supplementary Figure 9. 2D-clinorotation and orbital shaking enables low-sedimentation within low-volume reaction vessels. a) Schematic comparison of the reaction vessels placed in static condition, in a 2D-clinorotator or in an orbital shaker. Cross sections of the embedded TXTL reactions display probable molecular paths in each condition. b) Bacteriophage T7 cell-free synthesis comparison between 1-g conditions and orbital shaking at 60 RPM. Two different TXTL batches were used for this experiment. Batch A corresponds to the same batch used for the clinorotation experiment. Batch B is a different batch, which explains the differences in PFU yield. c) Statistical summary table for the shaking control experiment.

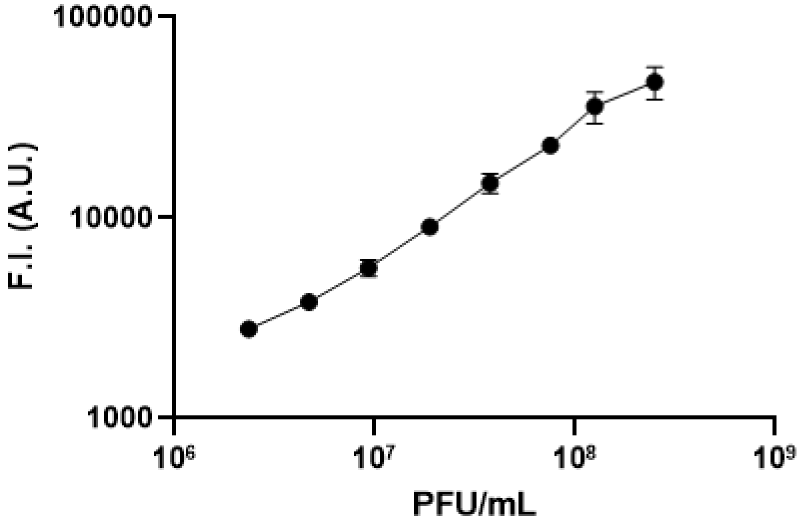

Supplementary Figure 10. Calibration curve for the dot-blot experiments. Pre-determined concentrations of T7 bacteriophages produced in TXTL systems were used to establish optimal scanning settings for the PFU/mL measured in the synthesis kinetics.

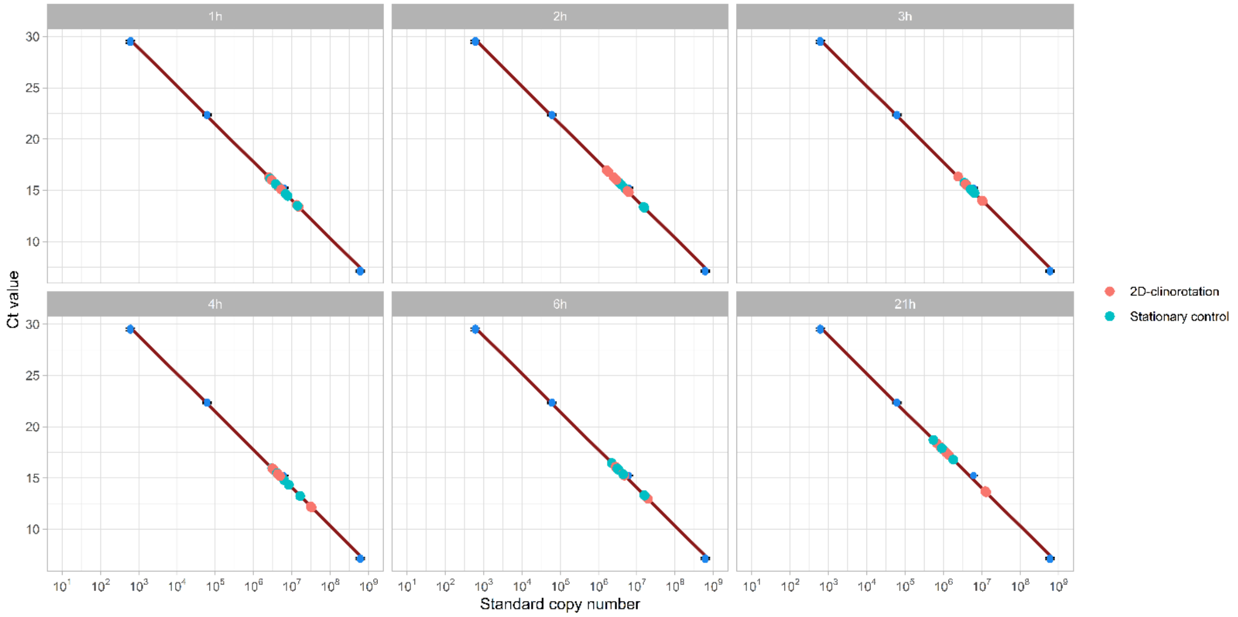

Supplementary Figure 11. The RT-qPCR results per timepoint projected onto the standard curve. Plotted are the total copy numbers in 5 µL of 100x diluted TXTL samples. The results were then corrected to 20 µL of the total reaction from which the DNA copy number per mL was determined.

Supplementary Table 1. List of T7 phage proteins detected by mass spectrometry and their associated statistics. Log2ratio, p-Value and q-Value correspond to the difference of averaged intensities peaks of the samples in s-μg compared to 1-g. The colormap of log2ratio is centered on 0 (negative values are red and positive values are blue).

Protein name	Abbrevation	Protein description	log2ratio T7 s-μg	p-Value T7 s-μg	q-Value T7 s-μg
P03726	EXLYS_BPT7	Peptidoglycan transglycosylase gp16	0.09410517	0.619589159	0.829362696
P03725	GP15_BPT7	Internal virion protein gp15	0.211401056	0.237367394	0.583862348
P00573	RPOL_BPT7	T7 RNA polymerase	0.059783722	0.75954832	0.898736323
P00581	DPOL_BPT7	DNA-directed DNA polymerase	-0.00902532	0.922500358	0.970855339
P00969	DNLI_BPT7	DNA ligase	-0.049660159	0.685584248	0.854089292
P03748	FIBER_BPT7	Tail fiber protein	-0.042447949	0.863543454	0.944713949
P03747	TUBE2_BPT7	Tail tubular protein gp12	0.051979995	0.730727255	0.882677549
P03696	SSB_BPT7	Single-stranded DNA-binding protein	-0.033412415	0.765950785	0.902556252
P03692	HELIC_BPT7	DNA helicase/primase	0.034877351	0.820116661	0.933229546
P03787	V5557_BPT7	Fusion protein 5.5/5.7	0.015180704	0.905155552	0.963883461
P00806	ENLYS_BPT7	Endolysin	0.204159787	0.019846392	0.420239352
P03728	PORTL_BPT7	Portal protein	0.108383002	0.403621646	0.686987295
P03694	TERL_BPT7	Terminase, large subunit	0.120603168	0.656456394	0.841631632
P03786	Y47_BPT7	Protein 4.7	0.274465461	0.203623083	0.551173505
P00513	PK_BPT7	Protein kinase 0.7	0.091480446	0.745839376	0.89052877
P03716	SCAF_BPT7	Capsid assembly scaffolding protein	-0.107561677	0.497981473	0.747876316
P00638	EXRN_BPT7	Exonuclease	-0.050901179	0.582761387	0.802891979
P03724	GP14_BPT7	Internal virion protein gp14	0.189530578	0.137178708	0.50423504
P03781	NUCK_BPT7	Nucleotide kinase gp1.7	0.351786207	0.038665058	0.47079694
P03797	Y38_BPT7	Protein 3.8	-0.033445295	0.796411635	0.921129341
P03785	ITAS_BPT7	Inhibitor of toxin/antitoxin system	-0.024237527	0.899720733	0.960540744
P03693	TERS_BPT7	Terminase, small subunit gp18	-0.06368776	0.828420896	0.936881863
P03793	Y16_BPT7	Protein 1.6	0.092167856	0.670571337	0.849237179
P03750	Y7_BPT7	Protein 7	-0.444467689	0.169306805	0.517799877
P00641	ENDO_BPT7	Endonuclease I	-0.136896019	0.283079282	0.608539939
P03704	VRPI_BPT7	Bacterial RNA polymerase inhibitor	0.295345968	0.149202798	0.510033585
P03800	Y65_BPT7	Protein 6.5	0.053760279	0.829980386	0.936881863
P03796	Y77_BPT7	Protein 7.7	-0.072891012	0.526687254	0.768103799
P03746	TUBE1_BPT7	Tail tubular protein gp11	-0.013333543	0.835764217	0.936881863
P19726	CAPSA_BPT7	Major capsid protein	-0.084937177	0.688752183	0.855459168
P03723	GP13_BPT7	Probable scaffold protein gp13	-0.013554018	0.913590507	0.967689824
P03803	SPAN1_BPT7	Spanin, inner membrane subunit	0.329015148	0.220874041	0.566006835
P03795	Y28_BPT7	Protein 2.8	0.015069037	0.92989573	0.974006114
P20406	GP59_BPT7	Probable RecBCD inhibitor gp5.9	0.015859888	0.844823203	0.939308852
P03784	Y43_BPT7	Protein 4.3	0.568749349	0.01470882	0.409374313
P03798	Y53_BPT7	Protein 5.3	0.246981903	0.061591431	0.4787412
P03751	GP73_BPT7	Protein 7.3	0.553323332	0.105829791	0.493904652
P03780	GP12_BPT7	Inhibitor of dGTPase	-0.090927084	0.620417839	0.829362696
P03802	HOLIN_BPT7	Holin	0.224738299	0.413478971	0.691585669
P03788	SPAN2_BPT7	Spanin, outer lipoprotein subunit	0.142527365	0.72600417	0.8805728
P03778	Y06_BPT7	Protein 0.6B	0.02362296	0.961529344	0.981947004
P03783	Y42_BPT7	Preotein 4.2	0.957644976	0.006037242	0.327878033
P19727	CAPSB_BPT7	Minor capsid protein	0.164073009	0.174660113	0.518216805
P03801	GP67_BPT7	Protein 6.7	0.015852896	0.946346575	0.975595572
P03775	OCR_BPT7	Protein Ocr	-1.177699875	0.122367158	0.497137089

Supplementary Table 2. Contents of each RT-qPCR reaction.

Component	Volume
Luna® Universal qPCR Master Mix (2x)	10 μL
Forward primer (CCTCTTGGGAGGAAGAGATTTG, 10 nM)	2.5 μL
Reverse primer (TACGGGTCTCGTAGGACTTAAT, 10 nM)	2.5 μL
TXTL sample (100x diluted in nuclease-free water)	5 μL

Supplementary Table 3. The program used for the RT-qPCR to detect bacteriophage T7 genomic DNA in TXTL.

Temperature	Time	No. Cycles
**50 °C**	120 s	1x
**95 °C**	120 s	1x
**95 °C**	15 s	40x
**60 °C**	60 s	
**95 °C**	15 s	1x
**60 °C**	60 s	1x
**95 °C**	15 s	1x

Supplementary Video 1. Visualization of the liquid/air interface in a PTFE tubing placed in an orbital shaker set at 60 rpm. The liquid is composed of 0.63 M of potassium permanganate in water.
